# Food Contamination: An Unexplored Possible Link between Dietary Habits and Parkinson’s Disease

**DOI:** 10.3390/nu14071467

**Published:** 2022-03-31

**Authors:** Giulia Caioni, Annamaria Cimini, Elisabetta Benedetti

**Affiliations:** 1 Department of Life, Health and Environmental Sciences, University of L’Aquila, 67100 L’Aquila, Italy; giulia.caioni@guest.univaq.it (G.C.); annamaria.cimini@univaq.it (A.C.); 2 Department of Biology, Sbarro Institute for Cancer Research and Molecular Medicine, Temple University, Philadelphia, PA 19122, USA

**Keywords:** healthy lifestyle, pollutants, in vitro and in vivo model for PD

## Abstract

Importance of a healthy lifestyle in maintaining the population’s well-being and health, especially in terms of balanced nutrition, is well known. Food choice of and dieting habits could impact disease management, which is especially true for Parkinson’s disease (PD). However, nowadays, it is not that simple to maintain a balance in nutrition, and the idea of a healthy diet tends to fade as the consequence of a western lifestyle. This should not only be dealt with in the context of food choice, but also from an environmental point of view. What we put into our bodies is strictly related to the quality of ecosystems we live in. For these reasons, attention should be directed to all the pollutants, which in many cases, we unknowingly ingest. It will be necessary to explore the interaction between food and environment, since human activity also influences the raw materials destined for consumption. This awareness can be achieved by means of an innovative scientific approach, which involves the use of new models, in order to overcome the traditional scientific investigations included in the study of Parkinson’s disease.

## 1. Introduction

The maintenance of a healthy nervous system is strictly related to a balanced lifestyle, and the preservation of its structure and function is influenced by various intrinsic and extrinsic factors, which include nutritional intake. The role of the diet in preventing the development of neurological disorders is indicated in several studies [1]. Nutrients, such as vitamin B, C, and D, long-chain omega-3 (ω-3) fatty acids, folate, flavonoids, and specific food groups and beverage positively contribute to cognitive function [2]. Food awareness could be a successful choice in a society, where the advancement of life expectancy translated into a higher prevalence of neurological disorders [3].

**Figure 1:**
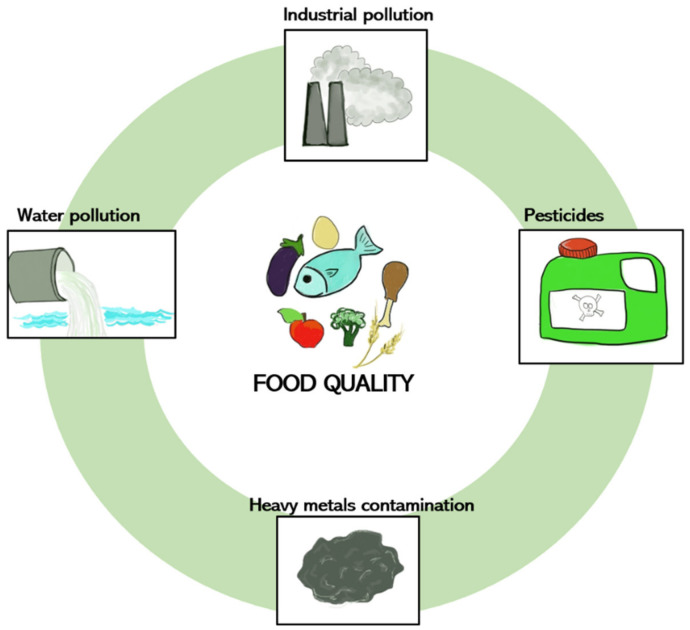
Impact of environmental pollutants on food quality.

However, when referring to food, it is not enough to merely gather information about nutritional values or properties. The assessment of food quality also involves the tracing of chemical food contaminants, which include unnatural substances (Figure 1). The most dangerous risks in the consumption of food are hidden and go beyond the consumer’s capacity to discriminate what is healthy or unhealthy. This concern arises from the man-made alteration of the environment, which contributes to the input of chemical compounds in soil, water, and air. Environmental contaminants may impact the food chain. Some of the best known examples are pesticides, which contaminate the raw sources of food, or heavy metals (copper, mercury or nickel) found in drinking waters or sediments [4].

Parkinson’s disease (PD) is a multifactorial disease [5], which derives from a combination of genetic and environmental factors. Whatever the causes, the hallmarks of PD include α-synuclein aggregation, lysosomal and proteasomal dysfunction, mitochondrial impairment, oxidative stress, and disturbance of iron metabolism [6]. These events lead to the progressive loss of dopaminergic neurons, while the surviving ones in the substantia nigra show intracellular inclusions known as Lewy bodies [7]. The underlying mechanisms of neuroinflammation are complex, and they also involve the participation of the cellular counterpart, such as microglia and astrocytes. However, the enzyme glycogen synthase kinase-3β seems to have a key role in inflammation [8], promoting the activation of microglia and the release of pro-inflammatory cytokines [9]. The involvement of glycogen synthase-3β in neurodegeneration was supported by many studies. For example, the inhibition of this enzyme could protect dopaminergic neurons from 1-methyl-4-phenyl-1,2,5,6-tetrahydropyridine (MPTP)-induced apoptosis [10]. Or even chemical inhibitors could have a neuroprotective function, attenuating the mitochondrial dysfunction mediated by the impairment of complex I [11].

Regarding the environmental causes, a more detailed investigation is necessary to explore sporadic (non-familial) forms of PD, about which many epidemiologic studies suggested a correlation with environmental factors [12,13]. The exposure to toxicants can occur in numerous ways and depends on the place of residence [14], occupation, habits [15], and also diet [16]. In this background, food can be considered a source of chemical compounds and toxicants, which could increase the risk of PD, particularly the onset of sporadic forms.

One of the main difficulties in studying sporadic forms of PD is related to the generation of suitable models capable of gathering all similarities with human disease. Many different in vitro and in vivo models have been used, including those generated through neurotoxins- or genetic-based approaches. Cell-based models are used for easy manipulation and the possibility to perform numerous assays. Animal models involve rodents, non-human primates, and non-mammalian species, such as zebrafish and invertebrates, including *Caenorhabditis elegans* and *Drosophila melanogaster* [17]. However, under the traditional approach, such models could not be appropriate to investigate the interaction between genetic and environmental factors.

For these reasons, it is necessary to investigate how dietary habits could expose the consumer to risk factors, evaluating the strict connection with environmental pollutants. It is important to point out the impact of chemical compounds deriving from human activities on food quality, raising awareness of environmental protection, not excluding human health. The assessment of the importance of nutrition requires new methods of investigation to overcome the limitations of traditional approaches, also placing an equally high priority on the multifactorial aspect of this disease.

## 2. The Impact of Food Contamination on the Onset of Parkinson’s Disease

Industrialization is responsible for the release of pollutants into the environment, which may negatively impact human life conditions. These chemicals influence the quality of soils, groundwater, also depositing on consumer products, especially fresh fruits and vegetables. Moreover, environmental pollutants released in the sea and freshwater can enter the food chain and be subjected to bioaccumulation phenomena [18].

There is evidence of pollutants responsible for neurotoxic effects, deriving from several studies conducted on animals as well as epidemiological investigations [15,19,20,21]. Here, the main contributors to food contamination are reported.

### 2.1. Metals

Metals and heavy metals can accumulate in edible foods, contributing to the increase in the exposure to these compounds. Heavy metal accumulation in food crops has been well-documented in Nigerian soil, for example [21], even if this problem also concerns the aquatic ecosystems [22] and sediments in other sites [23,24]. In this background, it is not difficult to predict that the probability to ingest contaminated food is really high, especially in particular geographical conditions.

#### 2.1.1. Mercury

One of the traditional examples is represented by mercury. Numerous studies investigated the presence of this metal in marine food, by sampling animal populations in the sea at particular latitudes [25,26,27]. Methylmercury bioaccumulation has severe consequences on human health, as history has taught us through the Minamata disaster (Japan, 1953). The Minamata disease is a kind of methylmercury poisoning derived from the ingestion of contaminated fish and shellfish [28]. This compound has been reported to induce Parkinson’s-like toxicity similar to 1-methyl-4-phenylpyridinium (MMP+) [29]. In fact, they both alter dopaminergic signal transduction, cause mitochondrial damage, and have consequences on neuronal pyruvate and propanoate metabolism, leading to energy deficit [29]. Moreover, in both cases, the neuronal damage and dopamine- and glutamate-related apoptosis are related to the presence of oxidative stress and high levels of oxygen reactive species [30,31]. A positive association between the consumption of contaminated marine food and the development of PD has been well-documented by Petersen et al. [32], whose study included 79 cases of idiopathic PD. The reasons beyond the higher prevalence of PD in the Faroe Islands have been investigated, and surprisingly, it was associated with the increased consumption of whale meat and bubbler in adult life [32]. The traditional diet is rich in predatory fish and mammals that feed on small invertebrates and fish. They have higher mercury concentrations than animals on the lower levels of the food chain [33].

#### 2.1.2. Manganese

Manganese (Mn) is essential to humans, since it is required for the development and function of the brain [34]. However, an excess in its accumulation may result in neurological alterations. In particular, in the 19th century, John Couper firstly described the symptoms deriving from Mn toxicity in employees working in grinding the black oxide of manganese [35]. He reported weakness, gait disturbance, and other symptoms similar to those observed in PD [36]. Detailed investigations on neuroplastic changes in the brain of welders exposed to Mn were presented by Chang et al. [37]. They examined the brain regions of 42 workers affected by chronic exposure to Mn, also revealing that the activation of cortical pathways could guarantee an adequate motor function with compensation of compromised regions.

The concern about occupational and environmental Mn exposure caused a major interest in the assessment of the related risks in Canadian welders [38]. Thanks to the studies carried out at the end of 1990s and in the early 2000s, welding operations were found to expose workers to a significant hazard, which could lead to neurological disorders and PD [38,39]. In particular, the detected concentrations of Mn during the assembly of large components were significantly higher than the threshold limit values defined by the American Conference of Governmental Industrial Hygienists [38]. The inhaled particles could reach cerebral sites, and Mn intoxication was shown to be associated with neurological symptoms [40].

The ingestion of Mn can occur through the consumption of contaminated food and drinking water. An epidemiologic study by Powers and colleagues (2003), conducted among 250 newly diagnosed PD patients and 388 control subjects showed an increased risk of PD, especially in those who combined the intake of Mn and iron [41]. In fact, these two elements was reported to have additive potential, causing together severe neurological alterations [42]. There are different kinds of food that naturally contain Mn and iron, including peanuts, beans, spinach, and peas. However, the contamination of soil, drinking water, and groundwater should be assessed to evaluate the hazard deriving from the ingestion of food.

### 2.2. Pesticides

As mentioned above, pesticides were reported to have neurological effects. Among pesticides, herbicides and insecticides are two different categories, which need to be discussed separately. Here, some examples are reported.

#### 2.2.1. Herbicides

Among the pesticides, the herbicide glyphosate deserves particular attention, because of its presence in food for animal and human consumption. The concern about the glyphosate derives from glycine similarity, because of which it is wrongly incorporated during peptides synthesis. Correlations between glyphosate and the increase in developing PD have been reported. Eriguchi and colleagues [43] reported the case of an atypical form of Parkinsonism in a young Japanese man with a history of glyphosate ingestion. Other studies described this form of glyphosate-induced Parkinsonism, but the exposure modalities include accidental acute skin [44] or long-term occupational exposure [45].

The mechanisms underlying glyphosate-mediated toxicity were also investigated in a rat model and involved glutamatergic excitotoxicity [46]. In particular, Cattani and colleagues [46] proposed the involvement of the activation of kinase cascades, the dysregulation of glutamatergic synapses and oxidative damage in rat hippocampus.

#### 2.2.2. Insecticides

A recent epidemiologic study by Kim et al. [47] showed the relation between exposure to insecticides at a tomato greenhouse and PD. In this study, they considered a proven case of occupational PD for the first time, rebuilding his family and occupational history and reporting the illness. Additionally, indications on environmental risk factors and substances used were shown. Many other studies focused on the role of insecticides in the onset of PD, and some authors explained the involved mechanisms of neurotoxicity. Organophosphates (OP) are just an example [48]. It is well known that Ops’ primary target is acetylcholinesterase, resulting in the alteration of cholinergic signaling. Moreover, the ability to increase reactive oxygen species was reported, along with the possibility to determine mitochondrial dysfunction with the generation of superoxides [49]. Different kinds of OPs could lead to PD-like symptomatology [48,50]. In vitro studies conducted on PC12 cells revealed that these could target the expression of PD-related genes [51]. Moreover, the PD risk seems to be influenced by the ability to metabolize OPs, depending on functional single nucleotide polymorphisms (SNPs) of the paraoxonase 1 gene [52].

### 2.3. Flame Retardants

#### Polybrominated Diphenyl Ethers

Polybrominated diphenyl ethers (PBDEs) are structurally similar to polychlorinated biphenyls, and they are used as additive flame retardants in the insulation of electronic equipment, in plastic polymers textiles, and furnishing. One of the most known commercial formulas is decabromodiphenyl ether (deca-BDE), which is added to polyester products, such as furniture upholstery or dense plastics [53]. It has been reported to be ubiquitous, since it can be easily released in the environment. Because of its chemical properties, such as lipophilicity, it tends to persist and enter the food chain, leading to biomagnification [54]. Human exposure to PBDEs occurs through diet, by ingesting contaminated food. The concern about these substances also derives from the neurotoxicological effects reported after pre- and postnatal exposure of mice and rats. In particular, long-lasting alterations in motor activity, learning, and memory were described [55]. The probability of ingesting contaminated food is not low. Some studies even reported the high contribution of seafood consumption and dust ingestion among countries, with particular attention paid to Korea [56]. The relationship between the exposure to PBDEs and PD is justified by the ability of these chemicals, at micromolar concentrations, to determine: high oxidative stress, impairment of calcium-dependent pathways, and inhibition of dopamine transporter (DAT) and vesicular monoamine transporter 2 (VMAT2) function. Thus, PBDEs seem to be specific for the dopaminergic system [54]. A similar concern was about the effects of exposure to polychlorinated biphenyls (PCBs), which can be ingested via contaminated food or dust. One of the major contamination incidents occurred in Belgium at the end of January 1999, when a mixture of PCBs accidentally contaminated materials destined for animal feeding [57]. These situations are extremely dangerous for the unaware consumer and make necessary the monitoring of pollutants. For example, in Italy, the Department of Prevention of the Local Health Authority of Taranto has monitored the dioxins and PCBs contamination in mussels since 2011 [58]. Although the production of PCBs and derivatives was banned many years ago, their contamination still remains a current problem [59,60].

## 3. Evidence and Mechanisms of Action

### 3.1. Metals

The concern about the exposure to metals developed when, in the past, some authors reported that the industrial use of heavy metals was associated with a major Parkinson’s disease mortality [61]. The hypothesis of occupational exposure to metals as a risk factor for PD was already introduced by Gorell et al. [62]. They explored the role of zinc, manganese, lead, copper, and also the association between different metals [62]. Metal ions seem to have an important role in the physiopathology of neurological disorders, resulting from post-mortem analysis of PD patients’ brains [63,64].

Mercury has neurotoxic effects [65], and the exposure can cause symptoms similar to PD. After mercury exposure or ingestion, a loss of dopamine receptors, tubulin and axon degeneration, glutathione depression, mitochondrial dysfunction, and tau phosphorylation were observed [66]. The neurological effects of mercury were firstly identified in the 1800s in hat makers, which demonstrated tremors, polyneuropathy, and other signs of movement disorders after mercuric nitrate intoxication [54]. Methylmercury poisoning determined parkinsonian-like symptoms, including alterations in movement and cognition, as observed after Minamata Bay’s disaster [54]. Even if it would be risky to correlate to PD [67], other studies demonstrated that methylmercury could influence dopamine metabolism in a manner similar to MMP^+^ [29,68].

The role of copper in the degeneration of the dopaminergic neurons is suggested by the presence of Parkinson-like motor-symptoms in patients affected by Wilson disease [69,70]. Wilson’s patients may show rigidity, resting tremor, bradykinesia, drooling, hypomimia, dysarthria, and gait disorders [71]. These manifestations are related to copper-mediated dopaminergic neurodegeneration in the nigrostriatal system [72]. Copper toxicity may result from chronic and long-term exposure to high levels of this metal and it may involve its accumulation in the brain. The sources are especially contaminated food and water [73]. In vivo experiments in the rat model showed apoptotic and inflammatory cell death in the corpus striatum with a decrease in dopamine and neuroprotective factors and superoxide dismutase 1 [74]. Copper mediates α-synucleins aggregation [75,76] and mitochondrial damage [77].

Among metals, the exposure to iron, manganese, and lead was recognized as risk factors for the development of PD and other related movement disorders [54].

Iron has biological functions essential for life (oxygen transport in hemoglobin, cofactor activity for cytochrome C and catalase); however, its accumulation in the basal ganglia was shown to be related to parkinsonian alterations [78]. As seen for copper-induced toxicity and Wilson’s disease, here too, the study of a pathology characterized by iron accumulation could open for further investigation. In this case, the hallmark of Friedreich’s ataxia is iron accumulation, and patients show motor dysfunction [79,80]. Free iron may be responsible for oxidative stress, which determines neuronal damage and, in general, neurotoxicity [54]. These considerations led to the hypothesis that even an external exposure to iron could have neurological consequences. In particular, some epidemiological studies evaluated the correlation between occupational exposure to iron via fumes coming from welding activities and dust from iron and steel production and an increased risk of PD [67]. However, several epidemiological studies did not give definitive answers. What is certain is that iron could have a role in the aggregation of α-synuclein, involved in the degenerative process of dopaminergic neurons [81].

Although it is required for the correct and normal functioning of many biological processes, manganese could have negative effects after its accumulation. Human exposure to manganese can occur through food, water, and via welding activities which contribute to its volatilization [54]; the inhalation of this metal has been associated with altered motor function and neurological disorders. A correlation between high manganese industrial emissions and an increased incidence of parkinsonian symptoms has been revealed, leading to a particular condition to which is referred as manganism [82], which shares similar mechanisms of degeneration with PD [82].

The human population is exposed to lead by means of food, drinking waters, dust, combustion of leaded fuels, and lead-based paint [54]. Fortunately, lead levels were reduced over the years, thanks to a policy of lead sources control and emission reductions. However, despite this, the risk is still present. Neurological symptoms related to lead exposure include peripheral neuropathy, ataxia, and motor abnormalities [83]. The first epidemiological studies seemed to be inconclusive, especially for the limitations of the methods used. For example, the assessment of lead blood concentration may not represent the best choice to investigate chronic exposure, since metals are quickly purged from the body [84]. Coon and colleagues [84] used an innovative method based on ^109^Cadmium excited K-series X-ray fluorescence to measure tibial and calcaneal bone lead deposition to determine occupational chronic exposure to this metal. The results showed an increased risk of developing PD [84].

### 3.2. Pesticides

Another cause of concern is represented by pesticides, since they are known to have neurotoxic effects [85]. Pesticides are consumed in large quantities, especially to keep up with the growing demands for plant products. It has been estimated that the worldwide consumption of pesticides in 2016 was about 3 million tons, and the main user was represented by China [86]. The extensive use of these chemicals can determine severe ecological effects, including biomagnification and bioconcentration [87]. Human exposure is frequent and occurs through the consumption of contaminated food.

Before epidemiological studies suggested the correlation with PD, the concern about the exposure to pesticides raised when the neurotoxic metabolite of MPTP, responsible for Parkinsonism in humans [88], was found to be similar to the herbicide paraquat, also exhibiting the same effects in animal models [89]. Epidemiological investigations showed that the occupational exposure to pesticides was linked to brain degeneration or nervous alterations [90,91,92]. More recently, different classes of pesticides and their neurological effects were investigated. Among the main pesticides known for having neurotoxicity effects, there are: paraquat, rotenone, maneb, benomyl, organophosphorus pesticides, carbamate, and mancozeb. They were usually identified as responsible for the onset of neurological disorders in agricultural workers, giving importance to the risks related to occupational exposure [93,94].

A recent study in Central California by Narayan et al. [95] provided evidence that the occupational exposure to carbamates, organophosphorus, and organochlorine pesticides increased PD risk, proportionally with years of exposure [95].

The mechanisms based on pesticide’s neurotoxicity are different and depend on the type. For example, the herbicide paraquat has been reported to cause nitrosative/oxidative stress [96]; the insecticide rotenone inhibits the complex I, leading to mitochondrial dysfunction [97]. Moreover, the fungicide benomyl inhibits aldehyde dehydrogenase, determining the increased production of the toxic metabolite 3,4-dihydroxyphenylacetaldehyde (DOPAL) [98]. The mechanisms of toxicity associated with organophosphates involve the inhibition of acetylcholinesterase, which causes the accumulation of the acetylcholine, responsible for the over-stimulation of cholinergic receptors [48].

Thus, the identification of environmental contributors to PD requires a deep knowledge of chemicals used in agriculture, industries and the monitoring of sediments, water, and soil. More generally, it may be necessary to carry out an assessment of environmental quality, along with raising awareness of this issue.

## 4. The Hypothesis of the Enteric Route: From the Ingestion to the Brain

A hypothesis beyond the development of the idiopathic form of PD was advanced by Braak and colleagues [99]. In particular, they postulated the possibility that a pathologic agent could penetrate inside the nervous system through the intranasal (via olfactory bulb) or enteric (via gut) route and be responsible for alterations in motility and non-motor consequences of PD [100]. Regarding the enteric route, Braak’s hypothesis would explain the spreading of the pathology from the enteric nervous system to the central nervous system through the vagal nerve and the dorsal motor nucleus of the vagus (in the medulla oblongata), and the affection of the lower brain regions towards the substantia nigra [101]. In this regard, several studies investigated the effects of oral or intragastric administrations of chemicals that are well known to induce PD-like symptoms. For example, in their study, Pan-Montojo and colleagues [102] showed that intragastric administration of rotenone in mice could determine the same symptoms and physiological alterations of PD. Another evidence of PD spreading from the gastrointestinal tract to the central nervous system is derived from studies on rats [103]. After the injection of α-synuclein, recombinant or deriving from PD patients’ brain lysate, this protein was transported—through the vagal nerve—to the dorsal motor nucleus of the vagus up to the brain. The mechanisms proposed could refer to the axonal transport mediated by microtubules [103].

More recently, Anselmi et al. [104] demonstrated that the administration of sublethal doses of paraquat along with lectin could induce alterations in gastric motility, which lead to Parkinsonism [104]. In particular, they observed the typical sign of α-synuclein aggregation in enteric and brain neurons, accompanied by the degeneration of dopaminergic neurons of substantia nigra pars compacta [104].

This kind of investigation is fundamental in the assessment of the risks deriving from exposure to environmental toxins. In fact, previous studies only focused on the occupational or disaster-related exposure. However, the same concern has to be addressed to contaminated food ingestion, even if the consumers have to deal with subthreshold doses. Environmental pollution involves all parts of the ecosystem, and necessarily humans, since we are the first users of raw materials. However, this also puts focus on the need to develop appropriate models in the evaluations of risks.

### Gut Microbiome in Parkinson’s Disease: Is It Susceptible to Pollutants?

Gut dysbiosis plays a key role in inducing central nervous system (CNS) neuroinflammation and neurodegeneration via the gut–brain ascending pathway. Devos and colleagues in 2013 showed in PD patients’ colonic tissue biopsies an increased expression of pro-inflammatory cytokines and increased activation of enteric glial cells [105]. Moreover, PD patients show a “leaky gut” which means an increase in the intestinal permeability and consequently gut dysbiosis that correlated with intestinal α-synuclein accumulation [106].

The dysbiosis increased intestinal permeability to bacteria and inflammatory bacterial products such as lipopolysaccharide (LPS) that in turn promote the disruption of the blood–brain barrier [107,108], facilitating neuroinflammation of the nervous system.

The maintenance of gut homeostasis is a dynamic interaction among the intestinal epithelial barrier, enteric neuro-immune system, and gut microbiota [109]. The enteric bacteria, which the main phyla are *Bacteroidetes*, *Firmicutes*, *Actinobacteria*, and *Proteobacteria* [110], contribute to the gut homeostasis interacting with epithelial cells, enteric immune system, and enteric nervous system.

The gut microbiota produce neurotransmitters such as serotonins, dopamine, and metabolites and can trigger immune cells to produce a panel of cytokines implicated in neurophysiology [111].

Although it is not possible to determine if alterations in the gut microbiota are a trigger or a result of PD pathogenesis, an altered microbiota is now a fact in PD patients [112,113,114].

Environmental factors, such as pollutants, including brominated flame retardants, high metals, and herbicides, produce dysbiosis, which in turn, might potentially lead to the establishment of a pro-inflammatory state in the gut and consequently impact on a wide range of human pathologies, including neurological disorders [115,116,117,118,119,120,121,122].

To date, it is evident that microbial transformation of ingested pollutants by gut microbiota can alter their toxicity and bioavailability [123,124]. For example, the flame retardant 2,2′,4,4′-tetrabromodiphenyl ether (BDE-47) caused a lower diversity, a community structure, and metabolic changes of the gut microbiota in a mice diet-induced obesity model [125]. Recently 2,4,4′-tribromodiphenyl ether (BDE-28) and perfluorooctanesulfonic acid present in the breastmilk of Norwegian mothers were correlated with a microbiota variety reduction, associated with less rich Veillonella and metabolism change, including a reduced concentration of acetic and propionic acids [126]. More interestingly, Scoville and Coll. [127] have shown that PBDEs’ impact on intermediary metabolites in an intestinal microbiome may contribute to PBDE-mediated toxicities [127], demonstrating the importance of microbiota and pollution interaction.

Recently, the study of the relationship between heavy metals and gut microbiota has improved considerably [128,129,130,131], confirming the complex interaction between the gut microbiota and heavy metals. It has been demonstrated that methylmercury alters the microbiota composition, inhibiting the growth of the *Lactobacillus* species (*reuteri*, *casei*, *acidophilus*). On the other hand, the microbiota produce hydrogen sulfide and hydrogen persulfide inactive methylmercury (MeHg) via the formation of sulfur adducts, thus decreasing its accumulation in organs such as the brain and liver [121]. Rat oral methylmercury administration changes gut microbiota and microbes’ metabolites, such as uric acid, xanthurenic acid, pyroglutamic acid, aspartic acid, serine, glycine, gamma aminobutyric acid (GABA), glutamate, leucine, and tyrosine [132], interrupting the above-mentioned bidirectional communication between the gut and CNS.

Data obtained on the effects of glyphosate on microbiota reinforce the observed link between intestinal dysbiosis and neurological disease [119]. The authors found a decrease in terms of *Lactobacillus* and *Bacteroidetes* bacteria. According to these data, a study has described that glyphosate inhibits the rate-limiting enzyme in synthesizing the aromatic amino acids tryptophan, tyrosine, and phenylalanine [133].

Overall, these data point to the existence of a bidirectional relationship between pollutants and gut microbiota. Perturbations of the gut microbiota by pollutants exposure may impact metabolic and physiological functions, contributing in part to the etiology or progression of neurodegenerative diseases.

## 5. Models to Study the Role of Nutrition in Parkinson’s Disease

Most of the information available on PD mechanisms derives from in vitro models. The main cellular sources include reprogrammed somatic cells from PD patients, genetically modified embryonic stem cells, neural progenitor cells [134]. Recently, human somatic cells, such as fibroblasts, can also be reprogrammed directly to induced neurons (iNS), using a combination of factors, which include myelin transcription factor 1 like (*Myt1l*), achaete-scute homolog 1 (*Ascl1*), and brain-specific homeobox (*Brn2*). At this point, the expression of forkhead box A2 (*FoxA2*) and LIM homeobox transcription factor 1 alpha (*Lmx1a*) could direct to dopaminergic phenotype [135]. A widely but less representative in vitro model is represented by the thrice-subcloned cell line derived from the SK-N-SH neuroblastoma (SH-SY5Y) cell line, a subline of the SK-N-SH cell line, which derives from a bone-marrow biopsy of metastatic neuroblastoma [136]. SH-SY5Y can be differentiated towards the dopaminergic phenotype using different protocols, among which the most used involves the use of retinoic acid. In this way, cells express the typical dopaminergic markers, becoming sensitive to dopaminergic neurotoxins.

Another method used involves the use of 12-O-Tetradecanoylphorbol-13-acetate (TPA), alone or in combination with the retinoic acid. PC12 cell line, which derives from a rat pheochromocytoma, is also a valid alternative. Having similarities with chromaffin cell characteristics, these cells share features with mature dopaminergic neurons [137]; in particular, the treatment with nerve growth factor induces the differentiation towards the catecholaminergic phenotype. To mimic the alterations determined by PD, drug treatment or genetic manipulation are commonly used. In the first case, the damage is induced by MPP+, 6-hydroxydopamine (6-OHDA), or rotenone; they are able to determine impairment in many cellular pathways, mitochondrial dysfunction, and oxidative stress [138]. In order to overcome the limitations related to in vitro models, several animal models have been used. Rodents, which include mice and rats, are extensively used: they are exposed to neurotoxic agents, such as 1-methyl-4-phenyl-1,2,3,6-tetrahydropyridine (MTPT), rotenone, or 6-OHDA in order to recreate nigro-striatal dopaminergic degeneration, which is also correlated to evident physical alterations [139].

Non-human primates and non-mammalian species were also used, and in particular, among the latter *Caenorhabditis elegans* and *Drosophila melanogaster* can be mentioned [139]. Genetically engineered models include α-synuclein transgenic rodents, characterized by the overexpression of wild-type or mutated form of α-synuclein [140]. They show the main signs of synucleinopathy, such as the accumulation of this protein in dopaminergic neurons or the formations of Lewy bodies, based on the type of promoter used to express the transgene [141]. However, there are no comprehensive studies based on the simulation of exposure to environmental pollutants, especially regarding the ingestion of contaminated food. Except for the investigations about the effects of intragastric administration of toxicants in rats and mice [102,104], PD models of chronic toxicant exposure usually do not involve the enteric route but modern devices. For example, the reproduction of PD phenotype in mice has been carried out using a subcutaneous implanted rotenone-filled mini pump, which could determine the chronic exposure to a certain dose of the substance [142].

### Zebrafish between Food Safety Research and Parkinson’s Disease Modelling

In recent times, zebrafish have also gained attention in neuroscience, representing a suitable alternative model to reproduce PD disease. It could be a useful tool to examine the mechanisms underlying neurodegenerative diseases, having the arrangement of the central nervous system similar to that of other vertebrates. Moreover, neuroanatomy has been studied and examined in detail in both larvae and adults [143,144]. From a genetic point of view, a zebrafish shares many human genes associated with neurological disorders. The other main advantages of using zebrafish in this kind of research are summarized in Table 1.

Regarding PD, zebrafish orthologs include the genes for several proteins, such as Parkin, DJ-1, PTEN-induced kinase 1, and Leucine-rich repeat kinase 2 [152]. In accordance with these considerations, transgenic models have been developed, using the morpholino injection for the knockdown of *dj-1* and leucine rich repeat kinase 2 (*lrrk*) genes, or transcription activator-like effector nucleases (TALENs) technique for PTEN-induced kinase 1 (*pink1*) [152]. The only limitation could be represented by the absence of ortholog to human α-synuclein gene, since only β- and γ-synuclein genes are expressed [153].

Neurotoxin-induced zebrafish models are also used, such as MPP+ models, which can determine locomotor alterations, movement frequencies, and a reduction in time spent moving, thigmotaxis [154]. MPP+ zebrafish models are able to recreate the motor symptoms of PD reported in humans, even if the non-motor alterations (including, for example, sleep or anxiety-like phenotype) cannot be reproduced [154].

In addition to being an attractive model from the neurological point of view, zebrafish have an important role in food safety research, since they allow an integrated approach to evaluate toxicological aspects related to the exposure to food contaminants [155]. Rodents present some management costs, ethical issues, and time-consuming experimental procedures and data processing limitations. The choice of using zebrafish is related to numerous advantages, including easy maintenance, easy manipulation and observation, for example, in the case of the fully transparent embryos. In addition, it can be considered as the “canonical vertebrate” [156], since it embodies the characteristics of this group, even from the genome point of view. For example, it is interesting to recall the high percentage (over 70%) of gene homology between human and zebrafish [146].

The wide use in food safety research is justified by the great similarity with the mammalian toxicity profiles, as reported in numerous studies [155]. Moreover, the administration of chemical compounds is really quick, and the procedure can also be standardized to ensure data reproducibility.

Many studies were conducted to test the innocuity of additives and preservatives, such as sodium benzoate [157], methylparaben [158], or nitrite [159]. However, the most interesting investigations refer to the effects of agrochemicals, which include pesticides [160] and herbicides [161], pharmaceutical residues, or heavy metals. Additionally, through zebrafish models, the correlation between certain pollutants and the PD-like phenotype has been reported even more.

Fenvalerate (FEN) is a pyrethroid insecticide, which is widely used in agricultural applications. At first, there was not a concern about its use, since it was considered less persistent than other pesticides and highly selective towards specific targets [162]. However, more recent studies underlined its presence in Chinese surface water, wastewater, and sediment, raising concerns about the possibility of bioaccumulation and biomagnification [163]. In fact, FEN has also been detected in human samples, such as breast milk [164]. These considerations determined the necessity to reconsider the effects of FEN on human health and also in terms of environmental pollutants. Among the toxic effects of FEN, neurotoxicity gained attention. Different studies have investigated the impact of the central nervous system and the involvement of pyrethroids in the onset of neurodegenerative diseases [165,166] with particular attention paid to PD-like symptoms [167,168]. In order to assess the neurotoxicity associated with the exposure to FEN, a zebrafish model was used, revealing the presence of PD-like symptoms in the larvae exposed [169]. This is also an example of the potentiality and the suitability of the zebrafish model, even in this field of application. In fact, FEN is one of the food contaminants the consumer could find in ready-to-eat salads, for example [170], or in the wheat destined to pasta production [171].

Some doubts could concern the macroscopic detection of PD symptoms in zebrafish models. Actually, motor symptoms are also easily recognizable in the teleost. Static tremors, postural disorders, and bradykinesia are just a few of the indicators used, and the other motility alterations or behavioral modifications can be studied and recorded.

**Figure 2:**
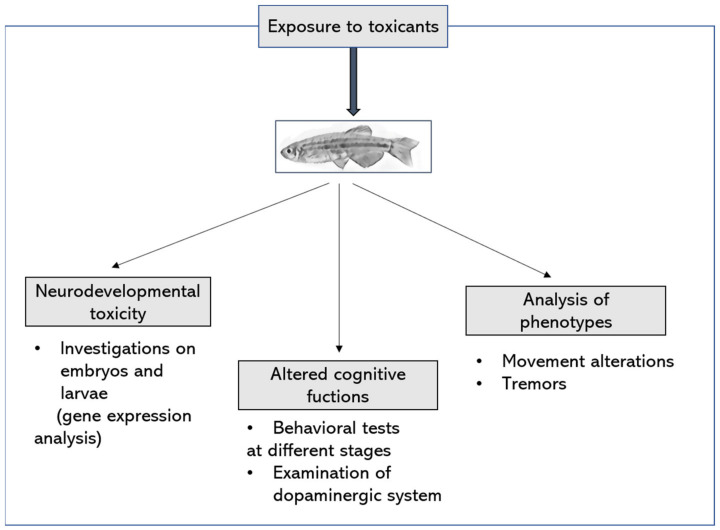
Example of experimental design and principal biological end-points after the exposure of zebrafish to environmental toxicants.

These considerations help to clarify the position of the zebrafish model in the research of neurological disorders and in particular PD. However, the great advantages can be related to the versatility of the model, even regarding the assessment of the damage. In fact, the experimental design and the definition of the biological end-points are flexible. The modalities of exposure to the toxicants can be organized in different ways to reproduce the exposure of humans to the same substances. Additionally, the consequent alterations can be revealed using gene expression or proteomic analysis, imaging, histological examination, behavioral tests, etc. Examples of end-points that can be considered are shown in Figure 2.

## 6. Conclusions

PD is a multifactorial neurological disorder, which may involve genetic but also environmental factors. The interplay between the external factors and the onset of neurological disorders is a recurring motif in the field of neurotoxicology. Industrialization has radically changed the quality of air, soil, and water, and the presence of chemicals is ubiquitous. The introduction of these substances in the environment directly influences the quality of ecosystems, but also indirectly human life. In fact, we draw from several sources, and each of them could determine food contamination. Water, sediments, air, and soil all determine the healthiness of food we ingest, and in turn, our health. The concern about pollutants exposure was raised when many studies hypothesized a correlation between the exposure to chemicals and the onset of neurological diseases, including PD. Actually, this relationship has been explored through epidemiological studies. Only a few PD models are able to recreate this kind of exposure. Hence, there is a need to develop reliable models which overcome the limitations given by traditional in vivo and in vitro models. For example, numerous advantages could derive from the use of zebrafish, which represents the canonical vertebrate, sharing similarities with human structures and physiology, through which the exposure to environmental pollutants could be mimicked.

## Figures and Tables

**Table 1:** Main advantages of using a zebrafish model.

	References
Easy maintenance, manipulation, and no time-consuming experimental procedures	[145]
Genomic homology with humans	[146]
Basic anatomical and physiological pattern conserved	[147,148]
Common molecular pathways	[149]
Fewer ethical issues	
Facilitation in drug administration (dissolution in water)	[147]
Faster determination of toxicological endpoints	[150,151]
